# Twenty-five years on: revisiting Bosnia and Herzegovina after implementation of a family medicine development program

**DOI:** 10.1186/s12875-020-1079-4

**Published:** 2020-01-13

**Authors:** Geoffrey Hodgetts, Glenn Brown, Olivera Batić-Mujanović, Larisa Gavran, Zaim Jatić, Maja Račić, Gordana Tešanović, Amra Zahilić, Mary Martin, Richard Birtwhistle

**Affiliations:** 1 Department of Family Medicine, Faculty of Health Sciences, Queen’s University, Haynes Hall, 115 Clarence Street, Kingston, Ontario K7L 3N6 Canada; 2 Department of Family Medicine, Faculty of Medicine, University of Tuzla, Tuzla, Bosnia and Herzegovina; 3 Department of Family Medicine, Faculty of Medicine, University of Zenica, Zenica, Bosnia and Herzegovina; 4 Department of Family Medicine, Faculty of Medicine, University of Sarajevo, Sarajevo, Bosnia and Herzegovina; 5 Department of Family Medicine, Faculty of Medicine, University of East Sarajevo, Foča, Bosnia and Herzegovina; 6 Department of Family Medicine, Faculty of Medicine, University of Banja Luka, Banja Luka, Bosnia and Herzegovina; 7 Department of Family Medicine, Faculty of Medicine, University of Mostar, Mostar, Bosnia and Herzegovina; 8 Department of Family Medicine, Center for Studies in Primary Care, Queen’s University, Kingston, Canada

## Abstract

**Background:**

The wars that ravaged the former Socialist Federal Republic of Yugoslavia in the 1990’s resulted in the near destruction of the healthcare system, including education of medical students and the training of specialist physicians. In the latter stages of the war, inspired by Family Medicine programs in countries such as Canada, plans to rebuild a new system founded on a strong primary care model emerged. Over the next fifteen years, the Queen’s University Family Medicine Development Program in Bosnia and Herzegovina played an instrumental role in rebuilding the primary care system through educational initiatives at the undergraduate, residency, Masters, PhD, and continuing professional development levels. Changes were supported by new laws and regulations to insure sustainability. This study revisited Bosnia and Herzegovina (B-H) 8-years after the end of the program to explore the impact of initiatives through understanding the perspectives and experiences of individuals at all levels of the primary care system from students, deans of medical schools, Family Medicine residents, practicing physicians, Health Center Directors and Association Leaders.

**Methods:**

Qualitative exploratory design using purposeful sampling. Semi-structured interviews and focus groups with key informants were conducted in English or with an interpreter as needed and audiotaped. Transcripts and field notes were analyzed using an interpretative phenomenological approach to identify major themes and subthemes.

**Results:**

Overall, 118 participants were interviewed. Three major themes and 9 subthemes were identified including (1) The Development of Family Medicine Education, (subthemes: establishment of departments of family medicine, undergraduate medical curriculum change), (2) Family Medicine as a Discipline (Family Medicine specialization, academic development, and Family Medicine Associations), and (3) Health Care System Issues (continuity of care, comprehensiveness of care, practice organization and health human resources).

**Conclusions:**

Despite the impact of years of war and the challenges of a complex and unstable postwar environment, initiatives introduced by the Queen’s Program succeeded in establishing sustainable changes, allowing Family Medicine in B-H to continue to adapt without abandoning its strong foundations. Despite the success of the program, the undervaluing of Primary Care from a human resource and health finance perspective presents ongoing threats to the system.

## Background

The wars that ravaged the former Socialist Federal Republic of Yugoslavia (SFRY) in the 1990’s were the worst to strike Europe since the Second World War. Triggered by declarations of independence by several of SFRY’s constituent Republics in 1991, the Yugoslav National Army, backed by surrogate armed forces in Croatia and Bosnia and Herzegovina (B-H), reacted with violence. After almost five years of conflict, an internationally enforced peace accord was implemented. By the time the Dayton Accord had come fully into effect in Feb.1996, B-H, a country of less than 4 million people, was left with an economy in tatters, at least 100,000 deaths, hundreds of thousands wounded and roughly half the population internally displaced from their rightful homes or driven into exile [1].

Under the terms of the Dayton Peace Accord, B-H was divided into two entities whose borders represented the territory occupied when a cease-fire was enforced: the Republika Srpska (RS), made up almost exclusively of Bosnian Serbs, and the Federation of B-H (FBH) consisting of an alliance of Bosnian Croats and so-called Bosniaks –primarily Muslim citizens or those of mixed ethnic or religious heritage. Complicating the picture was the creation of the independent condominium of Brčko District (BD), a multiethnic transportation hub, to be administered by an international supervisor. The capital city remained in Sarajevo but almost all authority for decision making lay within the entity structure, including health care. In the FBH this was further complicated by the delineation of 10 Cantons established along ethnic lines with most primary health care decisions to be made at this level. In RS, control remained more centralized in the Ministry of Health and Social Welfare in the capital of Banja Luka leading to a more uniform approach to health care service delivery.

This conflict set precedents for the wars that have followed with the deliberate targeting of civil, cultural, educational and medical facilities. The World Bank estimated that 60% of the houses in B-H, half of its schools and one-third of the hospitals and community primary care centers (dom zdravljas) were damaged or destroyed [2]. Many physicians and nurses were killed, wounded or fled to other countries. The education of medical students was profoundly affected, as was the training of specialists, leaving a traumatized, displaced population with greatly increased health needs and with fewer people to turn to for help, working under often impossible conditions. In a sense, this level of destruction provided an environment to explore structural changes to the pre-war health care system with its reliance on unsustainably large institutions, its fragmented primary care services, poor continuity and coordination of care and an ineffective gatekeeper function. For a more detailed description of the primary health care system and its challenges please see Additional file 1.

### Queen’s University Family Medicine Development Program in B-H

In the latter stages of the war, a health care reform commission in besieged Sarajevo began to plan a rebuilt post-war system founded on a stronger primary care model [3]. They were steered towards the systems of Family Medicine education and practice in several countries including Canada. At this time, a project led by Queen’s University in Kingston, Canada, for physical rehabilitation of war victims, provided initial contacts, facilitated through the local WHO office, leading to an exploratory visit by the Chair of the reform commission, the Dean of Medicine at University of Sarajevo, to the Department of Family Medicine at Queen’s University (DFM-Q) in the late winter of 1995. This resulted in six extensive missions to B-H over the following year by a senior faculty member of the DFM-Q (GH) who met with health leadership of all three ethnic groups and key international agencies to assess needs and potentials in the health care system and to establish trust. In March 1997 the Queen’s University Family Medicine Development Program in B-H (hereafter the Queen’s Program) officially began, with funding support from the Canadian International Development Agency (now Global Affairs Canada). Throughout three cycles of Canadian government funding to December 2005, the Queen’s Program also collaborated with the World Bank’s Basic Health Project and George Brown College School of Nursing of Toronto, developing and implementing a Program of Additional Training (PAT) for teams of practicing doctors and nurses [4]. This nationally standardized one-year program extended to all parts of the country, providing a rapid expansion of primary care reform based on a standard team model of Family Medicine. With support from the World Bank’s second primary health care project, the Health Sector Enhancement Project, Queen’s continued its work until Dec.2010 [5]. This fifteen-year bilateral relationship between a Canadian academic institution and the primary health care system of an entire country in the context of recovery from war while reinventing itself as a democracy, was unique.

Several main areas of focus were identified during the almost 2-year inception phase, working with leadership within the Ministries of Health, Medical Faculties and dom zdravljas. A continuum of educational initiatives, supported by new laws and regulations to insure sustainability, was developed, from undergraduate medical education, development of a Family Medicine specialization program (FMSP), additional training for teams of older physicians and nurses (PAT), to building of academic credentials through master’s and PhD programs [6–11]. This strategy had to be flexible, necessitating adaptation and shifting areas of focus in the unstable, post-war environment. (Table 1).

**Table 1:** QUFMDP Main Program Objectives

1. Founding of 5 academic Departments of Family Medicine (DFM) within Faculties of Medicine as central points for all teaching and research initiatives and to legitimize the new specialty.	
2. Reform of curriculum for medical students to strengthen the teaching of primary care knowledge and clinical skills. Standardized curriculum implemented in both entities with consistent teaching methods and content meeting European standards.	
3. Early recognition of education needs of primary care nurses leading to a parallel project funded by Canadian government and implemented in cooperation with George Brown College School of Nursing for training Family Medicine nurses.	
4. In cooperation with the World Bank, design and implementation of a nationally standardized one-year Program of Additional Training (PAT) for teams of practicing primary care doctors and nurses, using a model of team-teaching and to accelerate the numbers of trained Family Medicine professionals across all regions of the country.	
5. Development of a new specialization in Family Medicine that met European standards and embodying the principles of Family Medicine enunciated by the World Organization of National Colleges and Academies of Family Medicine (WONCA). This replaced the old specialization of General Practice taught exclusively by specialists in hospital settings. The specialization in FM was a major focus for the Queen’s program with specialization centers affiliated with each DFM, plus 2 satellite centers to accommodate demand.	
6. Support for the necessary credentialing of academic members of DFMs through completion of MSc and PhD degrees and for research and publication for academic promotion to leadership positions.	
7. Involvement as technical advisors for strategic planning, development of Ministerial and University policies and regulations, as well as Health System and Health Insurance Laws relevant to Primary Care/ Family Medicine.	
8. Because of long-term presence in country, acted as informal technical advisors/ facilitators/coordinators for many other NGOs, embassies, individual initiatives to leverage investment in health sector.	

One of the first strategies was to establish academic Departments of Family Medicine (DFM) in each medical faculty. In the absence of certified specialists with PhDs in the discipline, a necessary requirement for university positions, an innovative approach was taken, using the presence of a professor of FM from DFM-Q (GH) as the founding Chair for all but one of the DFMs. Potential future FM specialist-teachers were identified by dom zdravlja Directors, university and Ministry leaders and professional development activities were directed towards them while plans for FM specialization were underway. In parallel to this, modeled on experience from Canada and to provide future DFMs with a clinical base, Family Medicine Teaching Centers were created, from which much of the practical education of medical students, FMSP residents, PAT trainees and nursing students, would be provided. These Centers were located within selected dom zdravljas in those cities with Faculties of Medicine. A more patient-centered approach to care was to be modeled with new, evidence-based standards of practice for nurses and physicians, delivered in the familiar setting of the dom zdravlja. In the post-conflict development setting of B-H where numerous, often stand-alone pilot projects were the norm, this integration within the existing primary care setting was important for sustainability [12]. Please see Additional file 2 for a map of Bosnia and Herzegovina showing the entity boundaries between Republika Srpska and the Federation of Bosnia and Herzegovina, as well as the ten Cantonal boundaries within the Federation, and the location of Brčko District. The cities hosting Departments of Family Medicine and Teaching Centers are marked on the map for reference.

The development of the Program of Additional Training (PAT) in parallel with the FMSP, responded to the need to provide an immediate increase of care providers for the increased demand for health services. There was also a recognized need to facilitate connections to other European primary care networks, both as a peacebuilding strategy but also to enhance professional development. Using the College of Family Physicians of Canada for technical support, Family Medicine Associations were established in both the Federation of BH and Republika Srpska in Oct.2000, leading shortly thereafter to acceptance of B-H as a country member within the World Organization of National Academies and Colleges of Family Medicine (WONCA).

The early years of project activities coincided with the rapid expansion of internet resources. To take advantage of this in a setting with few local learning resources, computer literacy courses and a novel program in English for Medical Purposes were provided to all trainees. Workshops were focused on evidence-based medicine, research methodologies, quality improvement and the development of locally relevant practice guidelines. Within a short period of years, Family Physicians from B-H were actively publishing and fully participating in European medical conferences.

The ongoing political instability, profound population needs, and a weak economy created frequent challenges to planning and implementation of project activities. This necessitated a constant, on the ground presence as well as great flexibility on the part of the funding agency when areas of focus had to change in response to crises in one part of the country or events in neighbouring countries, such as the Kosovo conflict. At Queen’s University, an unprecedented thirteen-year secondment was approved for the senior Department member to live in Sarajevo and serve as Project Director (GH).

### Program outcomes in summary

After fifteen years of coordinated effort by B-H peoples at all levels, supported by a regular presence of Queen’s Program clinical educators, professors, nursing instructors and researchers, much was accomplished. This was made possible by Health Laws in both the Federation and RS that embedded Family Medicine in the health care system, regulations that defined Family Medicine specialization and the PAT, and the regulated approval of Family Medicine Associations [3]. (See Table 2 for a summary of these key accomplishments.)

**Table 2:** Summary of Selected Key Program Accomplishments

1. Twenty Family Medicine Teaching Centers established in the cities with Medical Faculties/ satellites, located in dom zdravlja’s [community primary care centres] as focal points for quality teaching and practice.	
2. Established FM Specialization program in 1999. More than 530 physicians certified as Specialists in Family Medicine by program end, with this number still growing.	
3. Five academic Departments of Family Medicine established in each of the Faculties, all being led by certified FM specialists with PhD / professorial credentials.	
4. New curricula in Family Medicine implemented for senior medical students consistent among all Faculties and meeting European transfer credit standards.	
5. More than 3200 practicing physicians and nurses trained under the Program of Additional Training, implemented with World Bank support in all corners of the country.	
6. Vibrant Family Medicine Associations established in each entity with 7 branches in Federation, including Brčko District, with membership actively representing B-H in significant numbers at European Conferences.	

### Research aims

The aims of this study were to attempt to understand the impact the Queen’s Program had on the education of medical students and family medicine trainees; the current state of academic family medicine and changes in the practice of primary care medicine eight years after the Queen’s Program ended. We also wanted to document ‘lessons learned’ in undertaking this long and complex international development project, lessons that might prove useful to development agencies working in the health sector, especially in conflict settings, and to those in the international donor community. It was apparent early in the planning stage of this study, that finding quantitative data from family practices, dom zdravljas and ministries of health would not be feasible. Therefore, this study was based on a qualitative assessment of the perspectives and experiences of health professionals, administrators and overseers in the primary care system.

## Methods

### Study design

This study adopted a qualitative exploratory research design. Our study was reviewed for ethical compliance by the Queen’s University Health Science and Affiliated Teaching Hospitals Research Ethics Board (file no. 6023423). Written informed consent was obtained from all participants.

### Setting

The Queen’s Program ended in December 2010. In May 2018, three Queen’s faculty (GH, GB and RB) returned to B-H to review the current state of Family Medicine and to assess the impact of the program’s interventions. Individual interviews and focus groups were conducted over a 2-week period in Sarajevo, Zenica, Mostar, Tuzla, Foča and Banja Luka.

### Participants and recruitment

We used purposeful sampling to recruit participants. This type of sampling was utilized in order to select information-rich participants that were particularly knowledgeable about or experienced with the phenomenon of interest [13, 14]. Additionally, purposeful, or purposive sampling, acknowledges the importance of the availability and willingness of subjects to participate, and their ability to communicate experiences and opinions in an articulate and reflective manner [15, 16]. This approach was selected in order to maximize the effectiveness of data collection given the limited time and resources allocated to the researchers during this period.

Participants were invited to participate in person or by telephone by a local research associate (LK) who also acted as translator and liaison between various groups and individuals in each city. Participants were eligible if they were involved in the Primary Care system in the study area and were willing and able to complete a semi-structured face-to-face key informant interview or participate in a focus group during the data collection period.

### Data collection

The interview guides for each group of participants (Additional file 3) were created prior to the visit and modified as new potential themes were identified. Interviews with participants were scheduled for one hour in length and conducted in Bosnian/Serbian/Croatian language or English and audiotaped for completeness. Each interview was led by one of the authors and notes were taken by all three researchers. An experienced translator with local and program knowledge was present for all interviews. After each interview potential themes were discussed, and questions were modified if new issues were identified. These new issues were incorporated into the final clusters of themes and subthemes.

### Data analysis

We used interpretative phenomenological analysis (IPA) to focus on the experience, understanding and interpretations of participants, to understand their views of the state of family medicine from a health system, practice and academic perspective [17]. In using IPA, the aim of the researchers was to explore the participants’ views and interpretation of an event in order to adopt an insider’s perspective, rather than creating an objective record of an event. As the research team was highly involved in the development and the implementation of the initiatives described in this article, using IPA allowed the researchers to recognize that their own conceptions are required to make sense of the experiences through a process of interpretive activity [17].

All notes taken during the interviews and focus groups captured the content and emerging themes and were reviewed by the authors (GH, GB and RB). Content analysis was conducted to identify emerging major and sub-themes by three authors (GH, GB and RB). One author (MM), who did not participate in the interviews, verified the themes by reviewing and auditing the codebook to ensure emerging themes were grounded and well represented in the transcripts and notes. Final themes were reviewed by four authors (GH, GB, RB, MM) to ensure consensus and inclusion of pertinent information.

## Results

Overall, 118 participants participated in the study, 75 from FBH, 46 from RS and 1 from Brčko District. Officials from the Federation Ministry of Health were not available. (See Table 3 for details on key informants and focus groups.)

**Table 3:** Interview and Focus Group Summary and Participant Composition

Region	Interview/FG #	# Participants	Group Composition
A	1	2	1 DZ Director and 1 Deputy Director
	2	4	1 DFM Head and 3 FM Specialists/DFM Members
	3	5	FM Specialists
	4	5	FM Residents
	5	1	Dean
	6	4	Medical Students
B	1	1	DZ Director
	2	2	1 DFM Head and 1 FM Specialist/DFM Member
	3	1	FM Association President
	4	3	FM Specialist
	5	8	FM Residents
	6	4	Medical Students
	7	1	Dean
C	1	2	1 Dean and 1 Vice Dean
	2	4	1 DFM Head and 3 FM Specialist/DFM Members
	3	4	Medical Students
	4	1	DZ Director
	5	4	FM Residents
	6	2	FM Specialists
D	1	1	Vice Dean
	2	4	1 DFM Head and 3 FM Specialist/DFM Members
	3	8	Medical Students
	4	4	FM Residents
	5	5	FM Specialists
E	1	1	Dean
	2	1	DZ Director
	3	5	1 DFM Head and 4 FM Specialist/DFM Members
	4	4	Medical Students
	5	4	FM Residents
	6	4	FM Specialists
	7	1	FM Association Specialist
	8	1	Accreditation Agency Director
F	1	1	Dean
	2	4	Medical Students
	3	1	DFM Head
	4	2	1 DZ Director and 1 Deputy Director
	5	1	FM Association President
	6	4	FM Specialists
	7	4	FM Residents
**Total Focus Groups/Interviews**			**39**
**Total Participants**			**118**
DZ Director/Deputy			7
DFM Head			4
FM Specialist			27
FM Specialists/DFM Members			13
FM Residents			29
Medical Students			28
Dean/Vice Dean			6
FM Association President			3
Accreditation Agency Director			1

Three major themes and 9 sub-themes were identified from the interviews. (Table 4).

**Table 4:** Major Themes and Sub-themes

*Theme 1 - Family Medicine Education Development:*	
Sub-theme 1a) Establishing Departments of Family Medicine	
Sub-theme 1b) Undergraduate medical curriculum change	
*Theme 2 - Family Medicine as a Discipline:*	
Sub-theme 2a) Family Medicine Specialization	
Sub-theme 2b) Academic Development – research and publication	
Sub-theme 2c) Family Medicine Associations	
*Theme 3 - Health Care System issues:*	
Sub-theme 3a) Continuity of Care	
Sub-theme 3b) Comprehensiveness of Care	
Sub-theme 3c) Practice organization	
Sub-theme 3d) Health Human Resources	

### Theme 1 - family medicine education development

#### Subtheme 1a): establishing Departments of Family Medicine

There was overwhelming support for the strategy of partnering with Universities and establishing DFMs at an early stage. This was key to the successful implementation of program objectives. The DFMs are seen as cornerstones for the development of the discipline and are looked to for teaching of all medical students, as the organizers and implementers of the PAT, and as trainers and examiners of Family Medicine residents and providers of continuing medical education (CME). Current department members were proud of their discipline and are committed to practicing and teaching its principles. They expressed that, undergoing FM specialization changed their perspective of themselves and their work, giving them a newfound sense of professional identity. There was consistent agreement that the knowledge base and clinical skills of family physicians in general have greatly improved.

Almost all DFM members were among the first generations of FM specialists trained under the Queen’s Program and see themselves as pioneers. However, this does place most of them in the same age bracket, causing some concern for succession planning in most Departments.

*” The biggest change was in our heads. We had a changed perspective of ourselves and our work, a sense of professionalism.” DFM member.*

All Directors of the dom zdravljas with teaching centers express pride in being the locus of training and have been supportive of consolidating and expanding teaching space as required.

The Medical School Deans express pride in the leadership of their DFMs and of the role played by DFMs in their medical curriculum and stated that, often, the DFM receives the award for best subject from graduating students. The Deans also acknowledge the key role played by the Queen’s Program in establishing the DFMs, developing and implementing the curriculum and changing how students were taught.

*“I am grateful for the curriculum Queen’s created here and for the active creation of the Department of Family Medicine here. We have not had to change the curriculum at all since you established it!” Dean of Medical School.*

There are still challenges in achieving equal status to other academic Departments in B-H. Although the other specialties are increasingly recognizing the skills and expanded scope of family physicians, DFM members at most schools still feel that their academic status is less than other specialties.

*"We need to fight for our positions" DFM member*

#### Subtheme 1b): undergraduate medical curriculum change

Integrating a core subject of Family Medicine into the final year of all medical school curricula was introduced by the Queen’s Program in the late 1990’s and was an early achievement for the DFMs. All 6 medical schools (a new school was opened in Zenica in 2016) continue to provide core teaching during the final year, based on the principles and methods introduced by Queen’s, adapted to meet European standards. Lectures are supplemented with supervised clinical work in a mentor’s practice. This “hands-on” exposure to patient-centered care, with responsibility for real patients with real problems was novel at the time of its introduction. Discussions with students indicate that other disciplines still do not provide a similar type of practical experience. Students are unanimously enthusiastic about the FM teaching they receive and, in all sites, described it as the best in their medical training. There was a broad consensus amongst Deans, family medicine faculty and students that mandatory earlier exposure to the principles of Family Medicine would be beneficial.

*“This was the best part of the whole curriculum! We gained a direct insight into how to work with patients and how the doctor-patient relationship is unique. Maybe make the first contact earlier in our teaching so we don’t discover it right at the end.” Senior medical student.*

### Theme 2 - family medicine as a discipline

#### Subtheme 2a): family medicine specialization

The FMSP continues to thrive in both entities with 107 residents (FBH 55, RS 52) currently in training.

The academic structure to the program continues to mature with relevant weekly academic seminars, resident scholarly projects and close FM mentor-learner supervisory relationships. The non-FM components of the specialization plan (e.g. in hospital speciality rotations) are not as well evaluated for their relevance and teaching, by both graduates and current residents. The concept of an academic year for all specialization trainees as an important human resource planning tool was dropped right after the Queen’s Program ended. Residents can begin training whenever approval is granted by the Ministry and the nationally standardized certification examination process has also been dropped, examinations being conducted at the local/Cantonal level.

The waiting period to get an approved FM specialization position can be as long as 7 years, this serving as a deterrent to graduating medical students and demoralizing to practicing doctors. The PAT has served as a stopgap for some Cantons in the Federation, successful completion reducing the length of specialization by one year. However, it has also had a negative effect on approval of specialization positions by Directors of some dom zdravljas, since a doctor who completed the one-year PAT versus a longer absence to complete the specialization program, can contract with the Health Insurance Fund to provide FM services.

Many residents stated that FM was not their first choice but, after waiting perhaps seven years for any specialization position they accepted it. Despite this, FM residents, their educators and FM Specialists all spoke of the value of their training, its focus on clinical and communication skills and its patient/family-centeredness as compared to the other sectors of the system. Providing continuity of care to the whole family is important to their professional identity and patients see them as being “their doctor” and as their trusted source for medical care. This was shared by all FM specialists interviewed.

*“Our patients know what we know, and they say: ‘Please don’t send me to someone else. Can’t you treat me’. This is a big change from the days when they just wanted me to refer them to someone else for tests.” FM Specialist*

#### Subtheme 2b): academic development – research and publication

Within the DFMs there has been strong commitment by members to obtain academic credentials and experience, all completing master’s degrees and many with PhDs, necessary for academic promotion. (Table 5).

**Table 5:** Academic Progress After FM Specialization

	FedBH	Rep Srpska
MSc (completed/in progress)	17	2
PhD (completed/in progress)	27	10

All DFMs are led by Family Medicine Specialists with PhDs and all department members are FM Specialists. This means that all FM teaching of medical students, residents and PAT trainees is done by competent, credentialed FMS physicians.

FM Specialists in BH actively participate in national and international conferences, publishing articles in Conference Proceedings and journals. This activity is not restricted to DFM members and is well supported by the FM Associations. Four DFMs have published textbooks in Family Medicine for their students and residents to use [18–21].

All of the Deans spoke highly of the academic place that Family Medicine holds in their medical faculties and also in the health care system.

*“Family Medicine is in a special position within our health care system because there are special people involved with it and it is growing.” Dean of Medical School*

#### Subtheme 2c): family medicine associations

There are well established Family Medicine Associations (FMAs) at the entity level with seven branches in the Federation, including Brčko District. Each has a President with a supporting executive, with affordable membership fees. Essentially all General Practitioners from the previous system, graduates from the PAT and FM Specialists can be members. The Associations serve as an umbrella organization for family medicine in their region and one of their prime purposes is to provide CME.

Branches have developed collaborative relationships with the medical schools, the Deans expressing respect for their work in organizing CME, annual Days in Family Medicine and regional and international conferences. These events are highly regarded by members with inter-entity activities being well attended. Brčko District has its own branch and plans activities with both the Federation and RS.

The Associations have engaged in practical measures to improve clinical care, assisting Ministries with development of Clinical Practice Guidelines. Compared to some other Specialist Associations, the FMAs are seen as cooperative and collaborative in their approach to achieving progress.

They have worked with the Physicians’ Union in wage negotiations and in discussions about public-private practice. They act as advocates in the policy and health insurance arenas as well as for individual members who require financial support to attend important European medical meetings, or after personal or professional troubles. The Associations have also played a key role in educating the public about the new discipline of Family Medicine, providing speakers for media panels or community health education events.

*“The Association has a vital role to work with the Ministry and the public. We are discussing important changes to the specialization curriculum to focus on enhanced skills which will improve quality of care and make Family Medicine more attractive to students.” FM Association executive member.*

### Theme 3 – health care system issues

An important program objective was to have the new certified FMS practitioners recognized as equals in the system and by the public, with incomes from the state competitive with other specialists. While there have been improvements in this perceived equality, there continue to be disparities in income and working conditions between family doctors and other specialists, primarily because of lack of private practice opportunities in Primary Care. Access to medical care is free to all citizens as are medications on an approved “essential” list for the elderly and disabled. Medication lists are decided at the Cantonal level depending on each Canton’s budgetary situation, the wealthier Cantons providing an expanded list of medications and diagnostic tests. Transferability of these benefits among Cantons is not always accepted. Because of its more centralized structure this is not a problem in Republika Srpska.

*“The status of FM specialists is lower according to many other doctors and the public. They just don’t understand what the FM specialists do. The public needs to be educated about the importance of the primary care system.” Senior medical student.*

#### Subtheme 3a): continuity of care

Access to a family physician (FP) is excellent with most patients seen on the same day if necessary. Appointment systems that were introduced by the Queen’s Program remain in place and mostly effective. The access to specialists is generally good and surgical wait times are reported as reasonable (e.g. 6 months for cataract surgery). There is also an organized system of home visits by both FPS and practice nurses. Our interviews suggested that there was now more continuity in the system; patients were less likely to ‘get lost’ and physician and patient satisfaction were much greater.

*“My patients now have excellent access, with good teamwork with my nurse. For patients with chronic conditions we can plan their visits in advance and the appointment system works well for them.” FM Specialist.*

#### Subtheme 3b): comprehensiveness of care

Family medicine teams are now providing more comprehensive care to families. School medicine (a separate service for care of school aged children) has been disbanded and now children over age 6 are seen by FPs. This was seen as a positive step by most because it allows for better continuity with the family but, by some, also seen as negative, because it increases the workload for an already overworked and understaffed sector.

However, FPs cannot practice to their skill level. Specialty-trained family doctors are skilled in diagnosis and management of both acute and chronic disease but continue to be hampered by outdated regulations. The ability to order some basic diagnostic tests (e.g. MRI, CT scan, thyroid testing, PSA or HbA1c) or to refer patients for endoscopy, prescribe insulin or screen women for cervical cancer is blocked by old rules that have not been re-examined, and these tests and referrals must be ordered or done by other specialists.

*“A cardiac ultrasound can take 5 visits, back and forth between the family doctor, general internist and cardiologist.” FM Specialist.*

Preventive health programs have become more common and this is seen as one of the strengths of primary care reform. There are now initiatives in cancer screening, smoking cessation, nutrition education and programs which focus on chronic diseases such as diabetes.

*“We have seen a 4% decrease in services provided per year because of improved prevention and education by FM teams.” dom zdravlja Director.*

#### Subtheme 3c): practice organization

Most FPs practice in dom zdravljas or neighbourhood-based ambulantas as salaried employees doing assigned shifts. They are paid by the number of patients rostered with no graded payment based on patient characteristics. Bonus incentives for meeting specific targets have been introduced in a few areas. Recently, there have been a few exploratory attempts to establish private clinics in some major cities but the regulation of these is yet to be worked out.

There is a shortage of available FM positions in most areas, making roster sizes untenable for practicing comprehensive care. FPs reported seeing 40–50 patients in a typical 7 1/2-h shift, but often up to 80 when short-staffed, which happens frequently. Roster sizes vary between 1800 and 5000, depending on Canton and city. Electronic record systems have been implemented but FPs report having to continue to complete a duplicate entry on paper. This is very time consuming and inefficient which adds to practice burden.

*“Our biggest problem is frustration with the system, and we are psychologically exhausted. We begin to lose our motivation and get suffocated.” FM Specialist and teacher.*

The medication prescribing process has improved. While a few other specialties working in community settings can prescribe, FPs still prescribe the majority of medications, often those recommended by consultants and not necessarily compatible with a patient’s other conditions, leaving FPs in difficult positions. Patients who require ongoing prescriptions can now get 3 months of medication at a time (rather than 2 weeks as it used to be) and don’t need to attend in person. E- prescribing is now well established in both entities.

#### Subtheme 3d): health human resources

While all education programs continue to be implemented, trainee numbers in the FBH are limited by Cantonal budgets. (Table 6) However, centralized human resource planning is not well implemented in the Federation, with decisions made at the Cantonal and dom zdravlja level. Positions are limited in both entities for new family doctors as are specialty training positions. When a physician is ill or on leave, absences must be covered by existing colleagues, further adding to their patient loads.

**Table 6:** Family Medicine Health Human Resources in BH

Federation BH 2001–2018			
Family Medicine Specialization Program Certificants			
2001–2010^a^		2011–2018	**Total**
340		137	477
Program of Additional Training (PAT) Diplomas			
2003–2010^a^		2011–2018	**Total**
Doctors	636	515	1151
FM Nurses	1434	606	2040
Subtotals	2410	1258	**3668**
Republika Srpska 2001–2018			
Family Medicine Specialization Program Certificants			
2001–2010^a^		2011–2018	**Total**
191		154	345
Program of Additional Training (PAT) Diplomas			
2003–2010^a^		2011–2018^b^	**Total**
Doctors	353	0	353
FM Nurses	783	321	1104
Subtotals	1327	475	**1802**
**Total FM** ^c^	**3737**	**1733**	**5470**
**Professionals**			
**Trained BH**			

^a^Under Queen’s Program direction and implementation. Numbers for PAT Federation do not include Doctors (101) and nurses (113) trained under the Swiss-funded project in Sarajevo Canton. Trainees from Brčko District are included in numbers for both entities

^b^ After 2011 Ministry of Health and Social Welfare in RS focused on PAT for nurses only

^c^ A further 143 nurses (FBH 85 and RS 58) were trained under a special program for nurses to work and teach in the FM Teaching Centers

A major concern expressed by many of those we interviewed is that many medical students are leaving the country for residency training elsewhere because of the limited opportunities both for specialty training and family practice, as well as for private practice options. The students we interviewed at some of the universities said that a majority of their class was considering leaving the country. Some also expressed a concern that, if they chose Family Medicine, they would not be able to control where they were assigned to practice, including being sent to rural areas with larger practice rosters.

*“Seventy percent of our class is studying German. We have to find some basic clinical work after graduation and even these jobs are hard to get. So, many will leave because they can’t even get to that first step! Sitting and not working for a year is unthinkable.” Senior medical student.*

An alternate view was expressed by a medical school Dean. “*Students leaving the country is nothing new for Europe with its open borders. It is healthy to get broader perspectives and see other systems. The trick is to get them to come back home after. It’s not a problem for them to go away but it is if they don’t return.”*

### Limitations

This study has several limitations. Due to time constraints in the data collection period, we were unable to get corroborating administrative or practice level data that would have strengthened our findings related to health system and practice related issues. We were unable to engage with officials from the Ministries of Health to obtain their views of the state of family medicine in B-H. However, we did gather some information from Deans of medical schools and dom zdravlja Directors. We also did not solicit views from other medical specialists or hospital administrators. We also did not interview patients of the system to seek their views. Selection bias may also have influenced the results as we used convenience sampling to select our participant group.

## Discussion

The overall finding is that the Queen’s Program has had a large impact on the organization and training of a workforce that is able to practice competent, comprehensive primary care. Academic family medicine is well established but still struggles to be seen as equal to the other established specialties. Where there has been less impact is in the work environment and in the scope of practice of family physicians.

All of the key program outcomes continue to develop, despite the challenging economic and political environment of post-conflict B-H. The strategy of setting an early focus on academic Departments of Family Medicine for sustainability has provided a solid educational foundation in all centers, providing high quality teaching that is valued and rewarded by students and Deans. A significant concern, however, is the factor of aging. Most members of DFMs were among the first generations of specialists and, except for a few Departments, there has been little promotion or preparation of a younger group as successors. This will need to be addressed as part of a wider primary care human resource renewal plan at a central, Ministerial level.

Medical students and family medicine residents are clear about the importance of family medicine in the system. However, despite the dramatic improvements in knowledge and skills of family physicians and their team nurses, status within the profession and to a lesser degree, in the public eye remains lower than for hospital-based specialties. This lower perceived status and lack of respect has been identified as a barrier to the delivery of primary care in other countries in the midst of rebuilding primary healthcare systems, such as in Serbia after the Kosovo conflict [22]. Students and residents also learn first-hand what a typical workday for a family physician looks like and this may serve as a deterrent to career selection. This phenomenon was also observed in a study looking at the introduction of an American model of primary care in the Russian far East, where it was found that excessive time demands on physicians contributed to preventing the development of an effective system of primary care [23]. The possibility of being sent to work in rural communities is also seen as a negative by students, as is the inability to establish a private practice, which would provide opportunities for higher incomes. But even for those who choose FM, a major obstacle is the lack of specialization positions. This is leading to a potential crisis as medical school graduates seek specialization positions elsewhere in Europe. Because of the practice environment, many who have completed specialization training in B-H are prone to leave the country in pursuit of better working conditions elsewhere. The challenges to retention of trained physicians has also been observed in other countries with newly created and reformed primary care systems, such as Ethiopia [24].

Roster size and daily patient volumes are the critical factors in this equation. Virtually every individual in every group spoke of this as the biggest issue to be solved if the true potential of family medicine is to be realized. In the specialist groups, rosters ranged from 1800 to over 5000 patients with an average of 2800. As was often said, these are not average, healthy citizens, but rosters of often chronically ill patients with high needs, a not unexpected impact of the conflict.

Dissatisfaction with the primary care system by the physicians in practice is not unique to B-H, nor is the falling interest in FM as a career choice, with many studies in recent years confirming the global nature of this problem [25]. In several instances, large roster sizes and work loads were noted as significant factors in both patient and physician dissatisfaction and declining interest among students [26–29]. Regardless, this is an enormous problem for primary care in B-H. Economic factors play a large role and the shifting of resources from other levels to increase the number of training positions and to at least slow the outflow of qualified physicians, has political implications.

A World Bank review of 2005 highlighted the “remarkable achievements” in family medicine development after the war but noted that the development of FM had “far exceeded” the rate at which legislative changes had been achieved and that there were no incentives to achieve a shift from secondary care to primary care. The report accurately predicted that the lack of reform at the secondary care level would make it difficult to achieve an integrated system with a true continuum of care [3].

*“The self-confidence and knowledge that we gained were very important so that we don’t blindly follow what some other specialist recommends and that we know is wrong. Our horizons were widened but now things outside Family Medicine must be improved. Patients get just as frustrated as we do.” FM Specialist.*

## Conclusion

Despite the impact of years of war and the challenges of a complex and unstable postwar environment, Family Medicine in BH has continued to adapt without abandoning its strong foundations. Academic Departments of Family Medicine, led by FM Specialists with PhDs and professorial rank, thrive at all medical schools in the country. All medical students receive an exposure to FM principles and practice that is consistently evaluated as excellent. The FM Specialization program has evolved and adapted to domestic needs and European standards. The discipline is well supported by active and respected FM Associations in both entities.

However, the successful implementation over the last twenty years is being threatened by an undervaluing of primary care from a human resource and health finance perspective and by the exodus of graduating students and younger physicians, untenable patient numbers and an aging generation of FM physician and nurse educators.

It is evident that there are not enough fiscal resources in both entities being dedicated to support a high-quality primary care system. This will require a renewed approach to health system planning which is more focused on primary care practice organization and delivery of care with other specialists in a consultant role.

## Supplementary information

**Additional file 1.** Primary Health Care System in Bosnia and Herzegovina

**Additional file 2.** Map of Bosnia and Herzegovina

**Additional file 3.** Interview and Focus Group Guides

## Data Availability

Data sharing not applicable to this article as no datasets were generated or analysed during the current study.

## Abbreviations

BD: Brčko District
B-H: Bosnia and Herzegovina
CME: Continuing medical education
DFM: Department of Family Medicine
DZ: Dom zdravlja, a community primary care health center
FBH: Federation of Bosnia and Herzegovina
FMA: Family Medicine Association
FMS: Family medicine specialist
FMSP: Family Medicine Specialization Program
FP: Family physician
IPA: Interpretative Phenomenological Analysis
PAT: Program of Additional Training
RS: Republika Srpska
SFRY: Socialist Federal Republic of Yugoslavia
WHO: World Health Organization
WONCA: World Organization of National Colleges and Academies of Family Medicine
