# Correction to: Twenty-five years on: revisiting Bosnia and Herzegovina after implementation of a family medicine development program

**DOI:** 10.1186/s12875-020-1091-8

**Published:** 2020-01-27

**Authors:** Geoffrey Hodgetts, Glenn Brown, Olivera Batić-Mujanović, Larisa Gavran, Zaim Jatić, Maja Račić, Gordana Tešanović, Amra Zalihić, Mary Martin, Richard Birtwhistle

**Affiliations:** 1 Department of Family Medicine, Faculty of Health Sciences, Queen’s University, Haynes Hall, 115 Clarence Street, Kingston, Ontario K7L 3N6 Canada; 2 Department of Family Medicine, Faculty of Medicine, University of Tuzla, Tuzla, Bosnia and Herzegovina; 3 Department of Family Medicine, Faculty of Medicine, University of Zenica, Zenica, Bosnia and Herzegovina; 4 Department of Family Medicine, Faculty of Medicine, University of Sarajevo, Sarajevo, Bosnia and Herzegovina; 5 Department of Family Medicine, Faculty of Medicine, University of East Sarajevo, Foča, Bosnia and Herzegovina; 6 Department of Family Medicine, Faculty of Medicine, University of Banja Luka, Banja Luka, Bosnia and Herzegovina; 7 Department of Family Medicine, Faculty of Medicine, University of Mostar, Mostar, Bosnia and Herzegovina; 8 Department of Family Medicine, Center for Studies in Primary Care, Queen’s University, Kingston, Canada

**Correction to: BMC Fam Pract (2020) 21:7**

**https://doi.org/10.1186/s12875-020-1079-4**

Following publication of the original article [1], the authors opted to correct the name of co-author Amra Zalihić from Zahilić to Zalihić. The original article has been corrected.
