# Supplementary material for: Twenty-five years on: revisiting Bosnia and Herzegovina after implementation of a family medicine development program
Source: BMC Fam Pract. 2020 Jan 13;21:7. doi: 10.1186/s12875-020-1079-4 (PMC6958717; doi:10.1186/s12875-020-1079-4)
Supplement: Supplementary file 1 — Additional file 1. Primary Health Care System in Bosnia and Herzegovina [file 12875_2020_1079_MOESM1_ESM.docx]

**Additional file 1 Primary Health Care System in Bosnia and Herzegovina**

Prior to the war in Yugoslavia, the health system was modeled somewhat on the Russian system which provided free access to primary care through community medical centres -Dom zdravljas (DZs) with a network of primary care clinics (ambulantas) but most of the diagnosis and treatment was provided by specialists. This was the system that we encountered in B-H after the war with general practitioners, who had no formal training in primary care, described as ‘traffic cops’ and nurses, with high school education only, working as ‘clerks’. With no appointment system nor established gatekeeper function, outmoded or missing equipment and facing crowded waiting rooms, the day consisted of directing patients to various specialists, completing forms for employer/government purposes, keeping cursory patient records and writing countless prescriptions or ordering tests on specialist recommendations. Nurses provided clerical services and carried out procedures on patients, often of questionable therapeutic benefit. The main goal of the entity Ministries, partnered with the Queen’s Program and other key donors such as the World Bank, the EU, Swiss Development Agency and WHO, was to establish a model of family medicine that provided the well proven features of an effective Primary Health Care system, with an emphasis on improving continuity and coordination of care and delivering a more evidence-based, affordable, expanded scope of practice. This was grounded in new Health Laws in both entities and expected standards for FM teams within Health Insurance Funds.^i-vi^

Atun RA, Kyratsis I, Jelic G, Rados-Malicbegovic D, Gurol-Urganci I. Diffusion of complex health innovations—implementation of primary health care reforms in Bosnia and Herzegovina. Health policy and planning. 2007 Jan 1;22(1):28-39.

Federal Ministry of Health. Strategic Health System Plan for the Federation of BIH. Bosnia and Herzegovina, Federation of BIH. Sarajevo. 1998.

Federation of Bosnia and Herzegovina. *Law on Health Care.* Official Gazette of the Federation of Bosnia and Herzegovina), No. 29/97. Sarajevo. 1997 Dec.

Federation of Bosnia and Herzegovina. *Law on Health Insurance.* Official Gazette of the Federation of Bosnia and Herzegovina No. 30/97. Sarajevo. 1997 Dec.

Ministry of Health and Social Welfare of Republika Srpska. *Law on Health Care.* Banja Luka. 1999 Jul.

Ministry of Health and Social Welfare of Republika Srpska. 1999. *Law on Health Insurance.* Banja Luka. 1999 Jul.
