# Supplementary material for: Twenty-five years on: revisiting Bosnia and Herzegovina after implementation of a family medicine development program
Source: BMC Fam Pract. 2020 Jan 13;21:7. doi: 10.1186/s12875-020-1079-4 (PMC6958717; doi:10.1186/s12875-020-1079-4)

**Additional file 2 Map of Bosnia and Herzegovina**

Entity boundaries between Republika Srpska and Federation of Bosnia and Herzegovina, and the location of Brčko District are marked. The six cities which host a Department of Family Medicine and Family Medicine Teaching Centers are also noted.


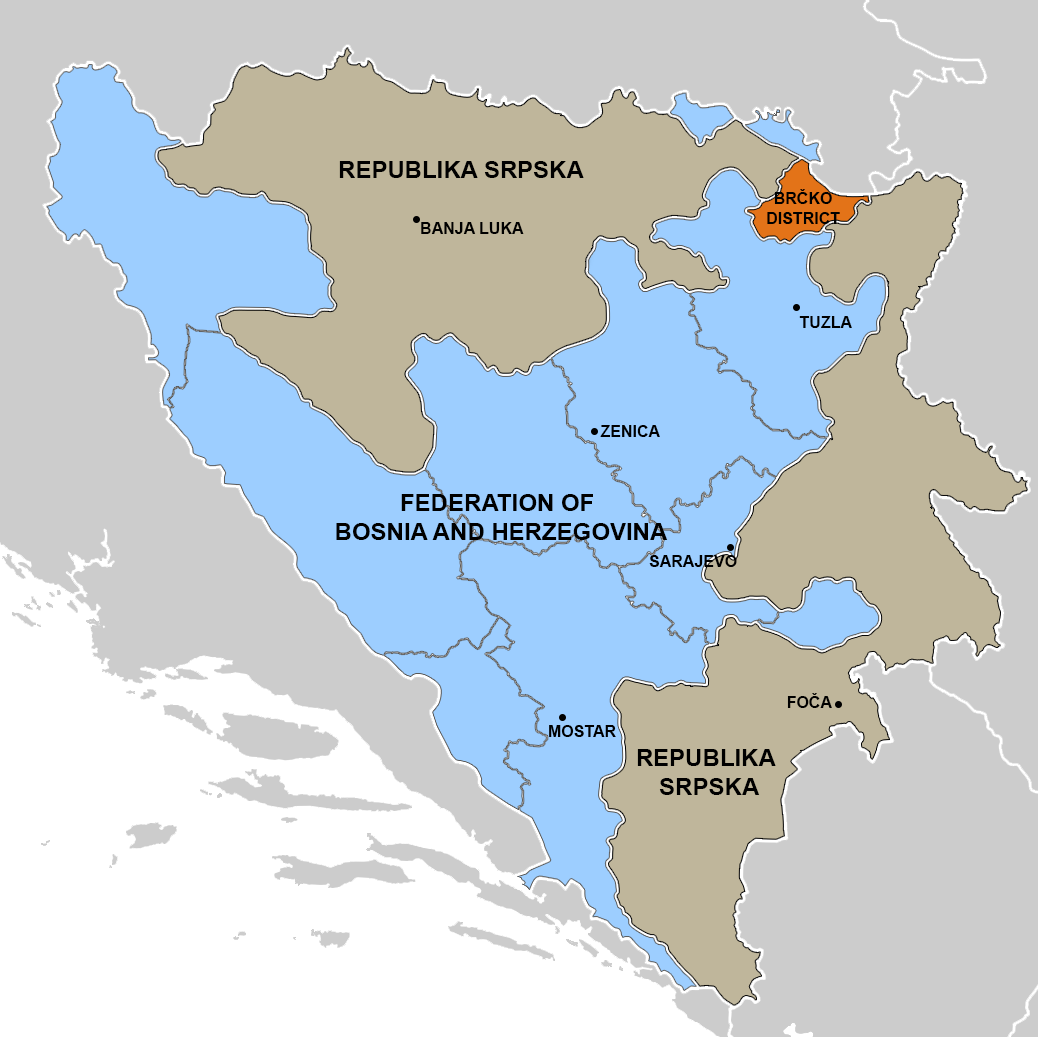

Supplement: Supplementary file 2 — Additional file 2. Map of Bosnia and Herzegovina [file 12875_2020_1079_MOESM2_ESM.docx]
