# Supplementary material for: Twenty-five years on: revisiting Bosnia and Herzegovina after implementation of a family medicine development program
Source: BMC Fam Pract. 2020 Jan 13;21:7. doi: 10.1186/s12875-020-1079-4 (PMC6958717; doi:10.1186/s12875-020-1079-4)
Supplement: Supplementary file 3 — Additional file 3. Interview and Focus Group Guides [file 12875_2020_1079_MOESM3_ESM.docx]

**Additional file 3 Interview and Focus Group Guides**

**Preamble (used with all participants)**

My name is [*insert name*] and I am [*insert position*] with the Department of Family Medicine at Queen’s University in Kingston, Canada. I will be leading the [*interview/focus group*] today and [insert name] will be assisting. The purpose of this research is to explore the impact of the Primary Health Care Family Medicine Development Program in the Balkans Region and the experiences of those who work in the Primary Health Care sector.

This [*focus group/interview*] will to help improve our understanding of any changes that have taken place since the introduction of The Program. It will take no more than 60 minutes. With your permission, it will be audio recorded. The recording will be used to ensure accuracy in the analysis of the data and will be destroyed once the project has been completed. Individual response will remain confidential and while I will use your name as often as possible in order that the transcriptionist can accurately capture participant input, any quotes used will be anonymized at time of analysis and/or writing. At any point, you may choose not to answer a question or to finish the [*focus group/interview*] discussion. Your participation is entirely at your discretion.

Any questions before we begin?

Please take a moment to read and sign the consent form if you have not already done so.

I am going to turn on the audio-recorder now.

**Item 1: Interview Guide – Medical Students**

**General and System**

1. Let’s start with introductions: Please state your name, title/position and the length of time you’ve held that position
2. How do you feel the Family Medicine system operates today?
   1. What are some of the strengths of the current system?
   2. What areas could be further developed to improve Family Medicine practice for your country?

**Health Policy and Family Medicine**

1. Please comment on the scope of practice of family physicians.

*Probes: has it expanded since before the program’s initiation? Can doctors order procedures and tests?*

1. How important do you feel family medicine is to the overall health of citizens?
2. What sort of skills do you think family physicians have in order to practice?

**Medical and Residency Education**

1. Do you see yourself training to become a family doctor?
   1. If no:
      1. What are some reasons you are not considering it?
      2. Are there any reasons you might be attracted to the profession?
   2. If yes:
      1. What are the reasons you are attracted to the profession?
      2. Do you see any drawbacks?
2. From your perspective, does Family Medicine receive adequate emphasis in medical school?
   1. Would you prefer more or less curriculum devoted to FM?
3. Did you spend any time in GP clinics as part of your training? Do you have any other clinical experience?
4. Do you feel your medical education is adequately preparing you for residency in Family Medicine?
5. Does certification in Family Medicine allow you to be seen as a “Specialist?”

**Final/follow up**

1. Are there any issues that you would like to comment on that we haven’t yet discussed?

**Item 2: Interview Guide – Residents**

**General and System**

1. Let’s start with introductions: Please state your name, title/position and the length of time you’ve held that position
2. How do you feel the Family Medicine system operates today?
   1. What are some of the strengths of the current system?
   2. What areas could be further developed to improve Family Medicine practice for your country?
3. How satisfied are you with the current system?

**Health Policy and Family Medicine**

1. Please comment on the scope of practice of family physicians.

*Probes: has it expanded since before the program’s initiation? Can doctors order procedures and tests?*

1. How important do you feel Family Medicine is to the overall health of citizens?
2. Do you see Family Medicine today in your region as:
   1. Accessible?
      1. If someone is sick, can they be seen today? Why or why not.
      2. What barriers may patients face when accessing Family Medicine in your region?
      3. What barriers do Family Medicine Physicians face when treating/accessing care for their patients? *Probes: test ordering, prescription drugs*
   2. Comprehensive?
      1. Are there limitations to treatments provided by Family Medicine physicians? Ex. Ordering routine blood tests, biopsies, small procedures, injections
      2. Is there an emphasis on screening for disease and/or preventative care? Is this seen as important? Why or why not.
      3. How important/comprehensive is the management of chronic disease (ex. COPD, diabetes, depression) in patients?
      4. Is there a long-term care system in place?
      5. Do physicians have the ability to visit patients in hospital?
      6. Please comment on current practices around End of Life Care and/or Palliative care. Is this seen as an important issue to patients? Physicians? Policy Makers?
   3. Able to offer Continuity of Care?
      1. Does the system support patients seeing the same doctor when they require medical attention/advice? (vs. the old model of doctors in ‘shifts’)
      2. Does the system support a ‘family medicine’ approach (where the whole family sees the same doctor)? Is there still an emphasis on children being treated by ‘school doctors?’
      3. Does the system take a longitudinal approach to care? Does the doctor see the patient from ‘cradle to grave’?

**Medical and Residency Education**

1. Did you choose family medicine as a career path?
   1. If yes:
      1. Why did you choose it?
      2. What are the reasons you were attracted to the profession?
      3. Did you have hesitations? Please explain these.
   2. If no:
      1. How do you feel now that you are studying to become a family doctor?
      2. Why weren’t you considering Family Medicine as a career path?
      3. Have your perceptions changed now that you’re studying (are you happy)?
      4. What, if any, are some appeals of becoming a GP?
      5. What, if any, are the drawbacks of becoming a GP?
2. What kind of emphasis does Family Medicine receive in medical school?
   1. Is there a specific curriculum?
   2. Are Family Medicine Departments adequately resourced?
3. Do you feel your medical education adequately prepared you for residency/practise?
4. Can you describe your work with mentors and other learning activities you have undertaken throughout your specialization?
5. Did you spend any time in GP clinics as part of your training? Do you have any other clinical experience?

**Final/follow up**

1. Are there any issues that you would like to comment on that we haven’t yet discussed?

**Item 3: Interview Guide – Graduates**

**General and System**

1. Let’s start with introductions: Please state your name, title/position and the length of time you’ve held that position
2. How do you feel the Family medicine system operates today?
   1. What are some of the strengths of the current system?
   2. What areas could be further developed to improve Family Medicine practice for your country?
3. How satisfied are you with the current system?

**Health Policy and Family medicine**

1. Please comment on your scope of practice as a family physician.

*Probes: has it expanded since before the program’s initiation? Can doctors order procedures and tests?*

1. How important do you feel family medicine is to the overall health of citizens?
2. Do you see family medicine today in your region as:
   1. Accessible?
      1. If someone is sick, can they be seen today? Why or why not.
      2. What barriers may patients face when accessing family medicine in your region?
      3. What barriers do Family medicine Physicians face when treating/accessing care for their patients? *Probes: test ordering, prescription drugs*
   2. Comprehensive?
      1. Are there limitations to treatments provided by family physicians? Ex. Ordering routine blood tests, biopsies, small procedures, injections
      2. Is there an emphasis on screening for disease and/or preventative care? Is this seen as important? Why or why not.
      3. How important/comprehensive is the management of chronic disease (ex. COPD, diabetes, depression) in patients?
      4. Is there a long-term care system in place?
      5. Do physicians have the ability to visit patients in hospital?
      6. Please comment on current practices around End of Life Care and/or Palliative care. Is this seen as an important issue to patients? Physicians? Policy Makers?
   3. Able to offer Continuity of Care?
      1. Does the system support patients seeing the same doctor when they require medical attention/advice? (do you have a defined practice population?)
      2. How well is the appointment system working?
      3. Does the system support a ‘family medicine’ approach (where the whole family sees the same doctor)? Is there still an emphasis on children being treated by ‘school doctors?’
      4. Does the system take a longitudinal approach to care? Does the doctor see the patient from ‘cradle to grave’?

**Family medicine and Medical Education**

1. Now that family medicine is a recognized specialty in your country, has this lead to improvements in the quality of care provided to citizens?
2. How well do you think your specialization training prepared you for practice?

**Final/follow up**

1. Are there any issues that you would like to comment on that we haven’t yet discussed?

**Item 4: Interview Guide – Deans**

**General and System**

1. Let’s start with introductions: Please state your name, title/position and the length of time you’ve held that position
2. How do you feel the Family Medicine system operates today?
   1. What are some of the strengths of the current system?
   2. What areas could be further developed to improve Family Medicine practice for your country?

**Health Policy and Family Medicine**

1. Please comment on the scope of practice of family physicians.

*Probes: has it expanded since before the program’s initiation? Can doctors order procedures and tests?*

1. How important do you feel Family Medicine is to the overall health of citizens?

**Family Medicine and Medical Education**

1. If you are able to, please comment on the changes you have witnessed in medical education in the past 20 years.
   1. What changes have made the largest impact?
   2. What areas could still be improved?
2. What kind of emphasis does Family medicine receive in your medical school?
3. Does your Department of Family Medicine have adequate resources? If not, what’s missing?
4. Are you satisfied with the representation of Family Medicine in the curriculum?
5. We understand that Family Medicine content is introduced during students’ last year of education. Can you explain the reasoning behind this? Are there plans to introduce FM curriculum during earlier years of medical study?
6. Is the current amount of time devoted to theoretical presentation and practical skills sufficient to prepare medical students for practice?
7. How adequately does the Family Medicine education program prepare students for future specialization or practice?
8. How are the department members supported professionally as teachers?
   1. What initiatives have been introduced to support faculty development and growth?
9. Do you see Family Medicine as a successful Career Path for medical students?
   1. Do you encourage students to pursue a career in Family Medicine? Why or why not?

**Final/follow up**

1. Are there any issues that you would like to comment on that we haven’t yet discussed?

**Item 5: Interview Guide – Association Leaders**

**General and System**

1. Let’s start with introductions: Please state your name, title/position and the length of time you’ve held that position
2. How do you feel the Family Medicine system operates today?
   1. What are some of the strengths of the current system?
   2. What areas could be further developed to improve Family Medicine practice for your country?

**Health Policy and Family Medicine**

1. Please comment on the scope of practice of family physicians.

*Probes: has it expanded since before the program’s initiation? Can doctors order procedures and tests?*

1. How important do you feel family medicine is to the overall health of citizens?
2. Have there been any changes to family medicine legislation or strategic plans that have an effect on family medicine practice?
3. From your perspective, what are the funding and support needs for family medicine including specialization or other training programs? Are the current supports for family medicine doctors adequate?
4. From your perspective, what are the main areas of responsibility of The Association?
5. What activities relating to Family Medicine has your association undertaken in the past few years?
6. What kind of communication systems are in place? Ex. Journals, newsletters, website, conferences
7. What formal affiliation agreements or relationships do you have? ex. WONCA Europe, SEE division

**Final/follow up**

1. Are there any issues that you would like to comment on that we haven’t yet discussed?

**Item 6: Interview Guide – DZ Directors**

**General and System**

1. Let’s start with introductions: Please state your name, title/position and the length of time you’ve held that position
2. Have you seen any changes in the organization of healthcare services in the past 20 years?
   1. Which ones specifically have caused the greatest impact?
   2. Which changes have been the most beneficial?
   3. Have there been any negative consequences as a result of changes?
3. How do you feel the Family Medicine system operates today?
   1. What are some of the strengths of the current system?
   2. What areas could be further developed to improve Family Medicine practice for your country?
4. What have you done to help further the development of Family Medicine?
5. How satisfied are you with the current system?

**Health Policy and Family Medicine**

1. Please comment on the scope of practice of family physicians.

*Probes: has it expanded since before the program’s initiation? Can doctors order procedures and tests?* *What are the status of the Work Medicine and School Medicine programs?*

1. How important do you feel family medicine is to the overall health of citizens?
2. Do you see family medicine today in your region as:
   1. Accessible?
      1. If someone is sick, can they be seen today? Why or why not.
      2. What barriers may patients face when accessing Family Medicine in your region?
      3. What barriers do Family Medicine Physicians face when treating/accessing care for their patients? *Probes: test ordering, prescription drugs*
   2. Comprehensive?
      1. Are there limitations to treatments provided by Family Medicine physicians? Ex. Ordering routine blood tests, biopsies, small procedures, injections
      2. Is there an emphasis on screening for disease and/or preventative care? Is this seen as important? Why or why not.
      3. How important/comprehensive is the management of chronic disease (ex. COPD, diabetes, depression) in patients?
      4. Is there a long-term care system in place?
      5. Do physicians have the ability to visit patients in hospital?
      6. Please comment on current practices around End of Life Care and/or Palliative care. Is this seen as an important issue to patients? Physicians? Policy Makers?
   3. Able to offer Continuity of Care?
      1. Does the system support patients seeing the same doctor when they require medical attention/advice? (vs. the old model of doctors in ‘shifts’)
      2. Does the system support a ‘family medicine’ approach (where the whole family sees the same doctor)? Is there still an emphasis on children being treated by ‘school doctors?’
      3. Does the system take a longitudinal approach to care? Does the doctor see the patient from ‘cradle to grave’?

**Family Medicine and Medical Education**

1. What is the place of the family medicine teaching centre in your DZ?

**Final/follow up**

1. Are there any issues that you would like to comment on that we haven’t yet discussed?
